# Identification of a clade A HIV envelope immunogen from Protocol G that elicits neutralizing antibodies to tier 2 viruses

**DOI:** 10.1186/1742-4690-9-S2-O7

**Published:** 2012-09-13

**Authors:** S Hoffenberg, S Kosakovsky Pond, A Carpov, D Wagner, A Wilson, R Powell, R Lindsay, H Arendt, J DeStefano, P Poignard, M Simek, S Fling, S Phogat, C Labranche, D Montefiori, D Burton, C Parks, C King, W Koff, M Caulfield

**Affiliations:** 1 International AIDS Vaccine Initiative, Brooklyn, NY, USA; 2 University of California San Diego, San Diego, CA, USA; 3 Duke University, Durham, NC, USA; 4 The Scripps Research Institute, La Jolla, CA, USA

## Background

Broadly neutralizing antibodies PG9 and PG16 have been isolated from the B cells of one clade A-infected individual from IAVI Protocol G. PG16 is relatively trimer-specific whereas PG9 binds trimer preferentially, but can bind monomeric gp120 from several viral isolates. Both antibodies are potent neutralizers that recognize greater than 70% of tier 2 pseudovirues in the TZM-bl assay. We sought to begin immunogen design efforts based on sequences from the Protocol G donor, however all viruses isolated from the donor were resistant to neutralization by PG9 and PG16. We used a bioinformatics approach to infer the most recent common ancestor (MRCA) sequence for the viral envelope (Env) to identify closely related viruses sensitive to PG9/16.

## Methods

Alignment of the MRCA sequence with 99 subtype A gp160 sequences from the Los Alamos HIV database identified BG505 as the virus with the highest degree of homology (73%) to the MRCA sequence.

## Results

Pseudoviruses prepared with this Env are sensitive to neutralization with a broad panel of bNAbs, including PG9 and PG16, indicating that BG505 has an antigen profile desirable in a vaccine candidate. When expressed as a soluble gp120 monomer from 293T cells, BG505 displayed a unique antigenicity profile – it bound well to both PG9 and PG16. We further show that a point mutation enables production of stable gp120 monomers that preserves the major neutralization epitopes on Env. Finally, we show that an adjuvanted formulation of this gp120 protein elicited neutralizing antibodies in rabbits (following a gp120 DNA vaccine prime) and that the resulting antisera compete with the bNAbs from 3 non-overlapping epitope classes for binding to gp120.

## Conclusion

The results indicate that BG505 Env warrants further investigation as an HIV vaccine candidate either as a protein or in a viral vector platform.

